# CRISPR-Cas antimicrobials: Challenges and future prospects

**DOI:** 10.1371/journal.ppat.1006990

**Published:** 2018-06-14

**Authors:** Elizabeth Pursey, David Sünderhauf, William H. Gaze, Edze R. Westra, Stineke van Houte

**Affiliations:** 1 Environment and Sustainability Institute, Centre for Ecology and Conservation, University of Exeter, Biosciences, Penryn, Cornwall, United Kingdom; 2 European Centre for Environment and Human Health, University of Exeter Medical School, Knowledge Spa, Royal Cornwall Hospital, Truro, Cornwall, United Kingdom; Geisel School of Medicine at Dartmouth, UNITED STATES

## Introduction

Antimicrobial resistance (AMR) poses a serious threat to modern medicine and may render common infections untreatable. The discovery of new antibiotics has come to a relative standstill during the last decade [1], and developing novel approaches to tackle the spread of AMR genes will require significant efforts in the coming years [2]. In 2014, several groups independently demonstrated how CRISPR-Cas (clustered regularly interspaced short palindromic repeats-CRISPR–associated), a bacterial immune system now widely used for genome editing, can selectively remove AMR genes from bacterial populations. Here, we discuss the current state of the field of CRISPR-Cas antimicrobials, the challenges ahead, and how they may be overcome.

## Using CRISPR-Cas to target AMR in bacteria

CRISPR-Cas is an immune system that protects bacteria and archaea against invading nucleic acids. Short sequences (‘spacers’) derived from foreign DNA or RNA elements, such as bacteriophages and plasmids, are inserted in CRISPR loci on the bacterial genome and later used by the Cas protein machinery to recognise and destroy invading nucleic acids carrying the same sequence. CRISPR-Cas systems are classified into two classes and six types, in which class 1 (types I, III, and IV) have a more complex architecture, with multiple Cas proteins participating in foreign DNA recognition and cleavage processes, whereas class 2 systems (types II, V, and VI) have simpler architecture, with recognition and cleavage carried out by a single multidomain enzyme. The latter class encompasses the type II CRISPR-Cas9 system, whose targeting specificity, versatility, and simplicity has led to many revolutionary applications in genome editing and ecological engineering. While most of these applications have been thoroughly reviewed [3], one that has received comparatively little attention is using CRISPR-Cas to eradicate AMR genes from bacterial populations and communities.

It was initially postulated several years ago that a synthetic CRISPR-Cas system could be utilised as an antimicrobial to kill specific bacterial genotypes [4]. More recent studies have confirmed the potential for CRISPR-Cas to precisely remove bacterial strains that carry genes, including those determining drug resistance, from populations and to re-sensitise bacteria to antibiotics by selectively removing AMR-encoding plasmids.

Highlighting the specificity of CRISPR-Cas antimicrobials, individual bacterial strains were selectively removed from a mixed population of *Escherichia coli* genotypes by transforming the population with a plasmid encoding CRISPR-Cas programmed to target a sequence unique to each genotype [5]. Two studies demonstrated that CRISPR-Cas9 can be delivered using phagemids (plasmids packaged in phage capsids) to selectively kill the clinically relevant bacterial pathogens *E*. *coli* [6] and *Staphylococcus aureus* [7]. One of these studies used phagemid transduction to deliver CRISPR-Cas9 constructs programmed to target AMR genes harboured on plasmids, which effectively removed these plasmids from bacteria. In addition, delivery of CRISPR-Cas9 by conjugative plasmids was used to kill bacteria carrying AMR genes in the chromosome [6]. The other study demonstrated sequence-specific killing of bacteria harbouring virulence genes using phagemid-mediated delivery of CRISPR-Cas9 and also showed that this approach was able to remove plasmids carrying AMR genes, thus effectively re-sensitising bacteria to antibiotics [7]. Both studies also showed that the CRISPR-Cas9 phagemids are able to kill specific bacteria in vivo, either in *Galleria mellonella* larvae exposed to enterohaemorrhagic *E*. *coli* [6] or on the skin of mice colonised with *S*. *aureus* [7].

While these studies showed that bacteria can be re-sensitised to antibiotic treatment using CRISPR-Cas, a clear problem was that these bacteria have no selective benefit over resistant ones, allowing residual resistant bacteria to be maintained in the population. In an attempt to increase the selective advantage of re-sensitised bacteria, a technology using temperate and lytic phage to re-sensitise bacteria to β-lactam antibiotics was developed [8]. In this case, CRISPR-Cas programmed to target AMR genes was delivered by a temperate phage. This CRISPR-Cas construct also conferred resistance to lytic phage, providing a subsequent selective advantage to re-sensitised bacteria that were challenged with this type of phage [8]. A further study implemented CRISPR-Cas9 for broad-spectrum targeting of common β-lactamase gene classes in *E*. *coli* by identifying a shared target sequence in >200 mutational variants of this gene [9], thus circumventing the problem of high sequence diversity among β-lactamase genes [10].

## Challenges ahead

### Complex microbial communities

Although CRISPR-Cas clearly has massive potential for the sequence-specific killing or re-sensitisation of AMR-carrying bacteria, at present, the use of CRISPR-Cas to remove AMR genes has only been assessed in near-clonal bacterial populations. Using such an approach in real-world environments, where bacteria are typically embedded in a microbial community [11], will be far more challenging. Natural microbial communities found within human, animal, and environmental microbiomes contain billions of cells per gram of matrix, consisting of thousands of species. Even within single species or strains, clonal lineages may possess different plasmids and other mobile genetic elements (MGEs) bearing diverse resistance genes. Quantitative PCR and next-generation sequencing allow quantitation and characterisation of bacterial hosts, MGEs, and resistance genes. However, determining resistance-gene carriage by specific bacterial hosts within complex communities requires more time-consuming approaches such as fluorescence-activated cell sorting of genetically tagged bacteria and MGEs prior to downstream analyses. Alternatively, recently developed methodologies such as epicPCR [12] and Meta3C [13] may be used to determine mobile gene carriage by specific host bacteria without the need for cultivation.

Another challenge associated with microbial communities is the difficulty in predicting community-wide responses to perturbations. Introducing CRISPR-Cas–based antimicrobials may have unwanted knock-on effects: If a strain is removed from a population, or its growth or metabolism is affected by removal of a particular plasmid, this may allow other, potentially more clinically problematic species to outgrow it. For example, it is well established that stress-induced alterations to microbial community composition and metabolite levels are associated with an increase in susceptibility to *Clostridium difficile* infection in the gut [14], and shifts in the species structure of the microbiome are linked with human disease states including diabetes and periodontitis [15]. The consequences of AMR removal by CRISPR-Cas in various microbial communities are to date unknown, and these risks must be assessed.

### CRISPR-Cas delivery vehicles and architecture

AMR genes are present in and spread amongst a wide range of bacterial species; they are frequently encoded on plasmids, which spread through horizontal gene transfer across diverse bacterial species [11]. Whilst phages can be powerful vectors for CRISPR-Cas delivery, the host ranges of most phage species are narrow, presenting an obvious barrier to targeting multiple bacterial species. Moreover, using this approach in spatially structured and complex microbial communities will provide an additional challenge, as the encounter rates between phages and their host bacteria will be reduced. Approaches to circumvent these challenges have been suggested, such as engineering phages to expand their host range [16, 17]; however, this technology remains at a preliminary stage. Another potential delivery vector is conjugative plasmids, which are transferred between bacteria, but restrictions such as narrow host range, barriers to plasmid uptake and establishment [11], and conjugation efficiency remain [6]. Finally, it is vital to consider the efficacy of various CRISPR-Cas systems in specific hosts, as cytotoxicity has hampered the effective use of Cas9 in some species. For example, in *Synechococcus elongatus*, a cyanobacterium, Cas9 is lethal when expressed constitutively [18], and attempts to use Cas9 in *Corynebacterium glutamicum* were also unsuccessful because of the toxicity of this nuclease [19]. However, in both of these cases, the alternative class 2 nuclease Cas12a was effectively used.

### Resistance evolution against CRISPR-Cas

Another issue is the evolution of resistance to CRISPR-Cas. In the context of CRISPR–phage interactions, this is known to occur readily through the acquisition of point mutations in the sequence targeted by CRISPR-Cas [20]. In principle, this could also happen in AMR genes that are targeted for removal, particularly if these AMR genes are under positive selection (e.g., when antibiotics to which the AMR gene confers resistance are used). Alternatively, resistance could evolve by inactivation of CRISPR-Cas loci through mutations or deletions in *cas* genes essential for target cleavage or by deleting targeting spacers [4, 21, 22]. Data from studies on CRISPR-Cas9–based antimicrobials suggest that mutations of target sequences are far less likely to occur than the delivery of defective CRISPR systems [6, 7]. Apart from the evolution of resistance through mutation, resistance can also evolve by selection for anti-CRISPR (*acr*) genes, which encode small proteins that bind to and inactivate critical components of the CRISPR-Cas immune system. At present, over 20 unique families of *acr* genes that target both type I and II CRISPR-Cas systems have been identified [23, 24]. Many of the Acr protein families targeting type I CRISPR-Cas systems have been identified in phages infecting *Pseudomonas aeruginosa* as well as other species within the proteobacteria. Although most of these Acr proteins appear to be specifically targeting one CRISPR-Cas subtype, one Acr has been identified that targets both the type I-E and I-F CRISPR-Cas subtypes [25], suggesting that more broad-range Acrs may exist. More recently, Acr proteins have been identified that target type II systems—which encompass the CRISPR-Cas9 systems used for gene editing [26]—one of which is notably broad in its target range [27]. The massive sequence diversity and high specificity of Acrs suggests that they are likely ubiquitous and possibly carried by MGEs such as phages and plasmids to circumvent targeting by CRISPR-Cas. Both the evolutionary consequences of CRISPR-Cas targeting of AMR genes and its impact on population dynamics require further study, especially using more realistic microbial communities to understand the ecological and evolutionary risks of this approach.

### Legislation and responsible governance of CRISPR-Cas–based antimicrobials

Upon development of a CRISPR-Cas system to remove AMR genes from environmental bacterial communities, the use of this technology would face a number of legislative and social issues. Exercising caution and fully assessing the risks when releasing or using gene-editing systems in the environment is essential. It has been suggested that there is a need for updated nongovernmental guidelines on the release of CRISPR-Cas and other gene-editing constructs that can be used to modify the genetic material of environmental populations, as well as a need for national and international bodies to develop guidance and legislation around the use of this new technology [28]. In addition to technical guidelines, community engagement is important, both to receive advice on best practices and gauge public and stakeholder support for the use of such technologies [29].

## Overcoming these challenges

**Fig 1:**
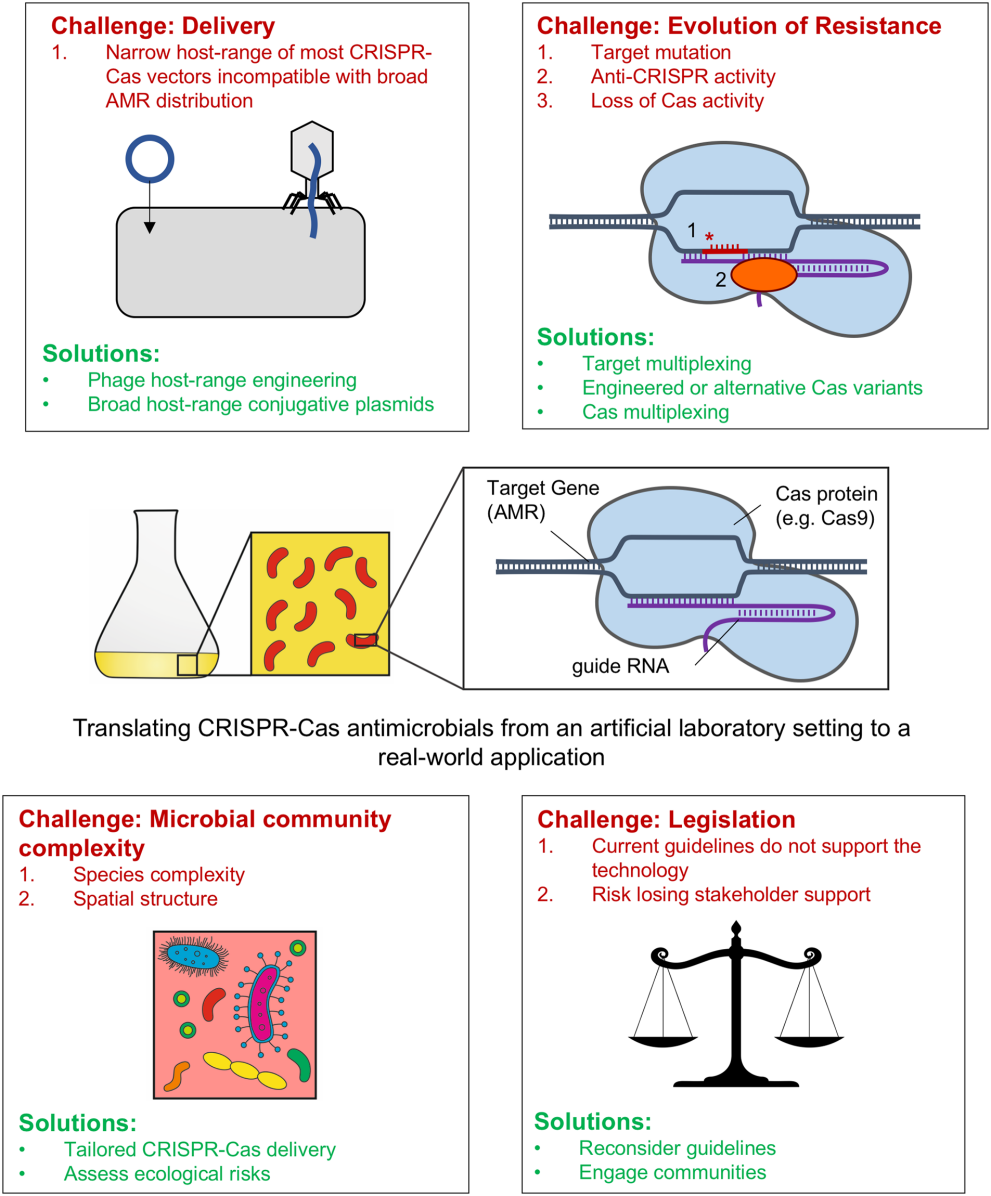
Challenges associated with CRISPR-Cas antimicrobials and potential routes to overcome them. A summary of the obstacles associated with using CRISPR-Cas–based antimicrobials in complex environmental populations of bacteria is shown. These include ensuring effective delivery of constructs (top left), routes of resistance evolution to these novel antimicrobials (top right), the species diversity and spatial complexity of bacterial communities (bottom left), and uncertainty in usage guidelines and stakeholder support (bottom right). AMR, antimicrobial resistance; CRISPR-Cas, clustered regularly interspaced short palindromic repeats-CRISPR–associated.

Although the obstacles to using CRISPR-Cas as a tool to tackle AMR are numerous, there are potential solutions to circumvent some of these challenges (summarised in Fig 1). The most pressing issue facing the use of CRISPR-Cas9–mediated removal of AMR is finding an appropriate delivery vector [6, 17] that is tailored for its particular purpose. For example, phage-mediated delivery may be the method of choice during an acute infection. However, even strains of the same bacterial species commonly vary in phage susceptibility, and alternative delivery vehicles may be desirable. Whilst their spread in microbial communities is slow, the wider host range of some conjugative plasmids would make them suitable candidates for use with a probiotic to protect against invasion by AMR-carrying bacteria or to remove microbiome-associated reservoirs of AMR. Although fitness costs associated with conjugative plasmids can limit their spread, these can be quickly offset by mutations in both the bacterial host and the plasmid [30–32], which could benefit the spread of a CRISPR-Cas–encoding plasmid in the community. The improvement of these tailored delivery systems would be a step towards tackling the challenge of the complexity of microbial communities, if a suitable broad host range vector could be identified or engineered.

Unpredictable ecological responses of a microbial community to the spread of CRISPR-Cas delivery vectors will be a major hurdle for the feasibility of using CRISPR-Cas antimicrobials in the real world. The ecological consequences of using CRISPR-Cas antimicrobials in a community context therefore need to be carefully studied by monitoring the effect of the removal of AMR genes on the frequency of other bacterial species and their associated plasmids.

Especially in the context of long-term applications, the evolution of resistance appears almost inevitable. However, resistance through mutation of target sequences can potentially be avoided by multiplexing, which involves CRISPR-Cas targeting of multiple sequences simultaneously to reduce the likelihood of resistance [33, 34]. The ease with which CRISPR-Cas (and CRISPR-Cas9 in particular) can be reprogrammed means this method can be adapted as needed and remains feasible. Selection for *acr* genes may be mitigated through using multiple CRISPR-Cas variants simultaneously, which would require different Acrs to escape targeting or engineering Acr-insensitive CRISPR-Cas variants. An approach using alternative nucleases to Cas9, such as Cas12a, may also circumvent any issues with the toxicity and efficacy of the system in various bacterial hosts.

## Future prospects

Many hurdles remain that need to be overcome before CRISPR-Cas can be used to target AMR in natural microbial communities. Identification of a suitable delivery method will be key to fully exploit the potential of this technology for limiting the environmental and clinical spread of AMR by MGEs. Simple reprogramming of CRISPR-Cas constructs to target particular genes of interest will greatly enhance the efficiency with which this can be achieved. Such an advance may have implications for tackling reservoirs of resistance and potentially help to retain or regain the antimicrobial activity of antibiotics. Future research is required to study and optimise the spread of CRISPR-Cas in more realistic microbial communities and to understand the risks associated with this technology. In addition, the social and legislative challenges associated with the widespread use of this gene-editing technology require active engagement with communities and development of clear guidelines to regulate its responsible and safe use. While using CRISPR-Cas systems that naturally occur on plasmids [35] may avoid some of the issues associated with the release of genetically engineered organisms, understanding the consequences associated with large-scale release of any DNA element will be key for sustainable and risk-free implementation of this technology.
